# Mobility Should Be Fun. A Consumer (Law) Perspective on Border Check Technology

**DOI:** 10.1100/tsw.2011.50

**Published:** 2011-03-01

**Authors:** Paul De Hert, Rocco Bellanova

**Affiliations:** ^1^ LSTS - Vrije Universiteit Brussels (Free University of Brussels), Belgium; ^2^ TILT- University of Tilburg, The Netherlands; ^3^ CReSPo - Facultés Universitaires Saint-Louis (Brussels), Belgium

**Keywords:** border controls, technology, body scanners, trusted travelers' programs, biometrics, Passenger Name Records (PNR), consumer law, data protection

## Abstract

After 9/11, states looked at transportation as if it was a matter of paying taxes: “We cannot make it fun, but we can make it efficient.” When traveling, we are asked to pass on data, give body samples, and pass through body scanners in the name of the general interest and in the name of our safety. Technology complements existing human checks and controls. Here we take a fresh look at the new security apparatuses and make transportation of humans more passenger-centered. Consumer protection law might help to complement the existing use of data protection law principles by citizen organizations. It should be possible to satisfy consumer needs, without forgetting the perspective of the citizen.

*"Get into the car*
*We'll be the passenger*
*We'll ride through the city tonight*
*We'll see the city's ripped backsides*
*We'll see the bright and hollow sky*
*We'll see the stars that shine so bright*
*The sky was made for us tonight"[1]*

## INTRODUCTION: PARADOXES AT WORK IN THE MOBILITY AND SECURITY DEBATE

There are many paradoxes surrounding technology, both in its development and its characteristics. In the case of border check technology, we shall single out two.

First, there is the "Heidegger's hammer paradox": we only see the hammer when it breaks. Technology and artifacts tend to reveal their nature only when they stop functioning. If they function well, we see them as part of our body. In fact, we are using the hammer and not the hammer itself. This is what we could call the problem of "low visibility".

Second, there is what one could call the “Dolly paradox”. Dolly was the first cloned sheep. Just her mere existence reminded everyone that cloning creates a genetically identical copy of an animal or plant. The paradoxical aspect here is that Dolly triggered a debate about cloning human beings years before such a thing was technically possible. This is what can be called the problem of “excessive visibility”.

Much remains unanswered about these paradoxical facts and the approach to technology that they subsume[2]. What is generating them? How do they come about? Can we influence them? And how can we avoid them in order to keep the ethical debate more serene?

The debates around the design and implementation of border check technology seem stuck in both paradoxes. At any rate, they create both a problem of low and excessive visibility complicating, to a serious degree, a rational debate on technology.

Now, let us turn to the object of our paper: a growing reliance on technology to organize mobility in general and at border checks in particular. The development from physical border checking to digital border checking is not uncontested. On the contrary, technologies such as body scanners, passenger data exchange, entry-exit registration systems, and biometrics are met with sentiments of upheaval and distrust.

The debates about these technologies might confuse some. Is it the shock of the new that inspires the critical voices or is there a general object of concern? If the latter is the case, what about the upheaval concerning more traditional border checking practices? Indeed, it has never been fun to cross borders, especially some decades ago in “Cold War Europe”; continuous checks at borders, huge discretion for border officials to select those that were subjected to intensive controls, and the existence of physical body checks to find drugs and other possessions. Was it really much better then compared to border checking practices that are at the design table or that are implemented today?

We believe that it would be unwise to downplay concerns about contemporary practices in the name of history and what can be remembered about it. At the same time, there is a strong case for giving new and developing practices at least some benefit of the doubt. Thus, only an open reflection about their possible use and the modalities of their use in contemporary societies can offer an opportunity to confront the two positions. And if this need is recognized, the ideal posture is the one that recognizes the challenges that technology creates without ignoring the new opportunities that they, actually or potentially, bring about with respect to the mobility of people, goods, services, and capitals.

Without pretending to be exhaustive, in what follows, we discuss some crucial aspects of the implementation of body scanners, trusted travelers’ programs, biometrics, and Passenger Name Record (PNR) data processing. All these technologies are examples of the deployment of a new mode of government of mobilities. This “technological mode” tends to operate by sorting individuals instead of countries; it widens the number of people submitted to control, downgrades nationality from the main criterion to one among many others, and classes individuals into categories with the help of risk-assessment procedures[3]. The deployment of this “technological mode” surely presents important challenges, not only in terms of privacy and data protection, but also in terms of nondiscrimination, presumption of innocence, and due process[4]. However, in this article, we look at these technologies from the perspective of the average citizen experiencing these technologies when crossing borders or attempting to do so. We conclude with a reflection on the added value of consumer (law) protection for the present discussions concerning data protection principles applied to security measures.

## BODY SCANNERS

Until the beginning of 2010, body scanner systems were mainly deployed in U.S. airports and mostly used as a secondary screening method[5]. Since then, other governments have decided either to increase their already existing stock, as in the case of Canada and Russia, or to introduce them, as has been the case of EU Member States. Among European countries, “formal trials” were conducted in Finland, the U.K., Netherlands, France, and Italy[6]. Finally, other governments have declared their intent to consider their introduction, as in the case of Australia, Japan, Nigeria, India, South Africa, Kenya, and, to a lesser degree, China and South Korea[7].

From the European level, the first discussions concerning the possible adoption of body scanners were linked to the presentation of the draft regulation on screening requirements for aviation security, proposed by the European Commission in 2008. In the draft list of allowed screening methods, the Commission first included the possibility of using body scanners for passengers. The main reaction was that of the European Parliament, who requested that the introduction of such a measure be avoided before the completion of a thoughtful impact assessment concerning its impact in terms of fundamental rights, health, costs, and benefits[8]. The Parliament also asked for the reports of other EU institutions and bodies, such as the European Data Protection Supervisor, the Working Group of the Article 29, and the EU Agency for Fundamental Rights[9]. The Commission finally decided to withdraw the relevant part of the draft regulation and launched, at the same time, a public consultation and a private-public dialogue that took the form of a “Body Scanners Task Force”[10]. Two years later, the Commission published a Communication on the “use of security scanners at EU airports” as a formal response to the 2008 Parliament Resolution, presenting the general and EU context of body scanners’ deployment, their use as security instruments, and the key issues raised by their introduction[11].

Again, without pretending to have a new, definitive perspective on this technology, we want to hint at some noteworthy facts[12].

First, there has been an astonishing debate about the added value of these technologies. Of course, anything that helps is at first sight welcome, but the simple question as to what these technologies are able to detect *more of* compared to other detecting technologies installed at airports is surprisingly left unanswered[13]. In the U.S., several actions under the Freedom of Information Act were necessary for the Electronic Privacy Information Center (EPIC) to obtain some (not all) of the technical data on this point from the U.S. Government[14]. It would seem to us that the question “Does this help?” is an important one, and that every bit of doubt or vagueness created will take away consumer trust.

Second, there is the lack of concern for consumer sensibilities with regard to bodily nakedness. These technologies had to be introduced from the start with imaging technology presenting the pictures as drawings of the persons under the scan. This feature is crucial and should not have been presented as a concession to data protection activism. The devil is in the details and one wonders if the vendors of these technologies and the governments and firms eager to deploy them ever cared for the details. The message seems to have been: “It is good for security, so go through it”. The more than evident question for consumers — “How should I go through it and what is the other guy seeing?” — should have been taken more seriously. Are images transformed in drawings? If yes, how easily can the person handling the machine go from the drawing to the real image? Also, if there are drawings, what is the level of abstraction? Why is a female drawn when female consumers pass through and why is a male drawn in the other cases? A simple abstract genderless figure would make a better case. In cases of doubt, abstraction could then be uplifted gradually. And finally, are the real images processed? And if yes, why and for how long?

Our Schiphol (Amsterdam) experience shows what can go wrong when haste is the rule. This Dutch airport, from a consumer perspective the most interesting (often in a negative sense), was one of the first in Europe to deploy body scanner technology. It did so for many reasons, a major one being their commercial willingness to stand out as the most American of the Continent. When we passed the body scanners, we noticed two important things. First, we were able to see the images processed by the machine in the screen of the operator and all the other passengers were able to see the same. Second, we saw that the drawings were much too similar to the people going through the scan. Even more, at the moment of writing this article, we do not recall whether there were drawings at all. Finally, the controllers were complaining about technical problems, the machines suggesting too swiftly that there was “something”, and thus pushing them to go to a higher level of scrutiny too often. So, even if plain old bodily nakedness was not as a default in theory, it was a default in practice.

We cannot but applaud the cautious approach of the European Commission, who took the time to better assess some of the key issues of these technologies before taking a stand on them[15]. However, we still regret a certain lack of precision on issues such as the efficiency and the effective added value of such machines[16].

Finally, it is interesting to note that, after a testing period, the Italian agency for civil aviation will probably abandon the introduction of body scanners in Italian airports[17]. According to the first interviews publicly released by the Italian experts, the two main reasons are the slow down of passenger processing at security controls and modest achievements in terms of efficiency[18].

The foregoing shows us that mobility policy making should move beyond vendor sales talk. The green light to new technologies can be given only after clear and transparent assessment, allowing the public at large to verify the arguments. A thorough privacy impact assessment, combined with a consumer assessment, is a prerequisite capable of gaining time rather than losing time. The assessment needs to be repeated and a green light should be necessarily revocable. If Schiphol or others deploy the machinery in a sloppy manner with unskilled or uncaring personnel, then actions need to be taken, including a stop order. Privacy violations need to be licensed under conditional terms.

## TRUSTED TRAVELERS AND FAST LANES

Now, let us turn to the introduction of “trusted travelers” programs, allowing prescreened, “trusted and registered” passengers to use fast-checking lanes. Within a market-driven system, there is nothing against making speed an asset to be paid for. Consumers understand price differences and product stratification. We know that business travelers enjoy other services and quality levels. We do not object, in principle, because in the market, there is a choice.

Something else, however, is at stake with the concept of “trusted traveler”. In the name of security, no such a thing can be considered. In the intelligence world, there is a system of trust levels, or security clearance, with thorough checks on peoples’ background that are a precondition to give them access to data or to specific communications. Of course, the system is not foolproof, and there is a constant danger for spies and corrupted officials to threaten the system. Outside the realm of government officials, the idea makes even less sense. In order to carry out one successful attack, it is sufficient that one person, trusted for decades, is threatened or corrupted into causing harm.

The Schiphol Privium system is not the only one of its kind, but it is a nice example of how things should not be done[19]. Schiphol, collaborating with intelligence services or police under unclear circumstances, pretends to be able to do just that: be capable of identifying trustworthy passengers who, after checks, become trusted travelers. Of importance is the claim that biometrics can help in this endeavor, by speeding the processing of travelers at automatic border gates. There, the iris of the passenger is scanned and compared to the data stored in the Privium card[20]. The same data are also accessible to the Dutch Border Police.

What the Schiphol example teaches us is that commercial motives and programs are free riding on the security rationale. Security is a service that is offered to distinguish between travelers and to create loyalty bonds with certain travelers that, supposedly, will continue to fly from Schiphol because of the privileges enjoyed[21]. We are only waiting for the next catastrophe caused by persons making intelligent use of this trust scheme. In the name of security, no such scheme is acceptable, whereas such a scheme is easily defendable in a normal commercial *rhetorique*. The damage to the biometric discussion is evident. The issue is infected with commercial strategies that harm a transparent discussion on the possible uses of these biometrics.

There are strategies to convince the European Commission to implement trusted traveler schemes all over Europe and we know that many EU officials, being frequent travelers themselves, are more than seduced by them[22]. From a consumer perspective, it would be wise to refrain from this commercial strategy that is sold as a security strategy. Unfair terms (a consumer law concept) are the only good qualification. The pressure on policy makers and air companies to uplift existing prohibitions on certain small objects (water, cosmetics…) and to streamline border checks for passengers at a reasonable speed shows that all consumers have an interest in speed and look critically at blocking mechanisms. One example of such a mechanism could be to put too few border and security officials on the normal lanes, forcing people, in practice, to enroll in the biometrically trusted traveler scheme to enjoy some comfort.

## BIOMETRICS

According to the online glossary of the European Data Protection Supervisor (EDPS), "biometrics or biometric systems are methods for uniquely recognizing humans based upon one or more intrinsic physical or behavioral traits[23]." Biometrics are not a novelty and have been used by government agencies since at least the 19th century, for both crime and population management[24]. However, as also mentioned in the EDPS presentation, their main present feature is linked to the mechanical capacity to read and process them, and thus the possibility to mechanically recognize human beings by comparison between the data collected and the data already stored. Then, biometrics are generally used for verification or identification purposes: either to validate the match of data between the carrier of a biometric document and the document itself, or to run the data of the individual against an existing database[25]. In the case of identification, a match between biometrics can lead to a further transmission of personal data.

In the context of border checks, biometrics are generally used at least in three ways. First, they can be incorporated into passports or other identity documents in order to "enhance" their security and fight against "identity fraud". Second, they can become part of a "traveler registered program" and used as a key, together with other documents, to pass automated gates. Finally, they can be collected at the ports of entry or at the moment of requesting visas or other entry permits, with the double purpose to create a database and to match new data against existing data, either in the same database or in other databases.

We have many questions about the way biometrics are implemented in the context of EU policy making. Large-scale biometric databases are created without transparent goal setting and proper discussions about risks. The technology is first tried out on criminals (national fingerprint systems), then on foreigners (Eurodac collection of asylum-seeker fingerprints), and finally applied to EU citizens in general (the EU passport allows for the storage of citizens' fingerprints). An evident consumer item is the price of these technologies and we expect developments in this regard when EU citizens realize that they will have to change passports at regular intervals and have to pay a considerable amount of money every time[26].

The general impression is of a biometrics policy dictated to the citizen without any choice. We contend: first, that there are possible uses for biometrics, but, second, that a more rational debate about error rates would take away some of the upheaval around biometrics. Let us start with the latter contention. Biometrics have an error rate. Depending on the kind of biometrics, this error rate is very small or relatively bigger. The problem with error rates is that even very small percentages do implicate many people when biometrics are used in a large-scale setting. To make things simple: an error rate of 0.1% might look modest, but if the intent is to control billions of people, then the error rate becomes more than small[27]. Does that mean that biometrics are unreliable? The answer fully depends on the expectations. In the fierce debates for and against biometrics, one party seems to argue that biometrics comprise a reliable technology, whereas the other party casts doubts. The answer seems to rely on the deployment of the technology with a "fallback" procedure that avoids the stigmatization of the individual mistakenly processed (or not at all recognized) by the control system, and that also grants him/her the choice to pass by a different control system. Some of our colleagues have reported on the Chinese use of biometrics at the border with Hong Kong. There, people can either choose a biometric border check or a physical one. The choice is open and there is no stigmatization of those who do not opt for having a biometric check. If a person undergoes a biometric check, then the goal should not be to detain this person or make him or her miss a train or a plane, but to complement the check with a traditional check without too much fuss. Granted, there is an error rate; these things happen and have to be anticipated.

The element of choice would also answer a wide set of concerns, ranging from religious and ethical concerns about inviolability of the body, to concerns of people who have no "clear" fingerprints because of their age or work, and also concerns about data protection. The choices regarding biometrics are manifold, but seldom properly identified[28]. The argument that Europe has no choice because the U.S. is pushing its own policies lacks credibility. The EU is imposing passports with fingerprinting on its citizens, whereas the U.S. is not doing the same with its citizens. There is no international law obligation to include fingerprints in EU passports. Iris and face biometrics could have done the job, but the EU has opted for more without a clear rationale. Security is not necessarily helped by this decision, since one could assume that if it did so, the U.S. would certainly have done so too.

## PASSENGER NAME RECORD DATA (PNR)

The debate about passenger data has been flawed by the same two issues: lack of choice and refusal to consider the consequences of the error rate of the profiling operations carried out on passenger data, in an endeavor to make the technology look perfect.

Let us first look at the issue of the error rate. Traditional law enforcement in the criminal area is centered on the notion of probable cause[29]. Without this probable cause or its EU counterparts, no search and seizure are allowed. The requirement protects against the so-called "fishing expeditions", but does not guarantee that innocent people are never the object of search and seizure. Accidents happen and miscarriages are part of the history of criminal law. There is no solid tradition of compensation in Western criminal law. The European Human Rights Convention, Article 5, foresees some compensation in cases of unjust physical deprivations, but does not foresee a broader right for compensation[30]. Again, a requirement such as probable cause is conceived to avoid mistakes as much as possible, contributing to a situation that, taken together, does not require the recognition of broader rights.

In the case of PNR, something completely different is at stake. The passenger data are not only required to verify a traveler's data with the names of persons on the no-fly list. Next to this simple identification scheme (already generating many mistakes), there is a twofold profiling scheme. On the one side, the no-fly lists are compelled on the basis of intelligence and profiling; while on the other side, the passenger data are used for both profiling purposes and profile generation[31].

We are very far from the traditional criminal law process. Errors abound when the way a passenger buys his or her ticket or chooses his or her meal on board might make him or her look suspicious. It goes without saying that the added value of this "guess work" has to be solidly proven. The consequences hamper mobility to an extreme degree. So far, there are little data about the added value of the PNR technology, although most law enforcement officials seem to be very confident about its success. What happens when one is put on a no-fly list by accident? There is an administrative procedure on the website of the U.S. Department of Homeland Security to report possible errors and some say that proper controls are then carried out[32]. However, this is far away from the notion of compensation that we have in mind. More should be done than just correcting data based on "guess work". Properly speaking, an excuse is to be expected coupled with a generous compensation for the harm suffered from being erroneously listed on a no-fly list. In a good consumer protection perspective, no evidence of harm needs to be given. There is distress and there are many anxieties about being refused to travel to a certain country, as well as for being singled out. A technology based on "guess work" accepts damage to individuals and should therefore not shy away when individuals ask for compensation. On the contrary, compensation should be built into the system *because* the system expects to work with errors.

The foregoing shows that there is an underestimated element of choice with regard to the PNR. In the present state of things, there is a choice that can be made for improving the system in the light of consumer interests. Technologies installed today are seldom considered in this light. Let us assume that critical analysis will show the efficiency of the PNR system in a couple of years. Assume the technology will remain. How many years longer will it take before making it more consumer friendly? Is there, for instance, a good reason to inform people that they are on a no-fly list at the very last moment when they are at the airport, in a hurry with their luggage, far away from their comfort zone? We do not see it. However, being on a no-fly list is public knowledge and you will eventually get to know it by simply trying to make it to a certain country and not being able to do so! So, why wait for the bad news? What needs to be established is a genuine consumer service: alerting the people that they are on a no-fly list so that they can cancel business meetings abroad and call their lawyer to start a verification procedure in case they think this is based on errors or identity swaps[33]. Law enforcement authorities such as Homeland Security should give access to their data when possible and when this is not detrimental for security purposes. An error based on a bad profile or an identity mistake should not be hidden under a veil of security interests, but should be confessed and compensated for properly[34].

## THE ADDED VALUE OF A CONSUMER LAW PERSPECTIVE

In this contribution, we have tried to think about mobility from a consumer perspective. That perspective is partly based on general assumptions about what consumers value. Of course, we all travel differently and our expectations are different. However, some expectations seem to be almost identical. Ryanair, for instance, is a low-cost carrier who claims to offer mobility at cheap prices, whereby “comfort is not to be expected”. In practice, this means that luggage, help with checking in, hunger, and thirst are needs or situations that will require extra money. Ideas for the future include passengers standing during the flight and paying for the use of the toilet.

Consumers then have a choice since they can fly other companies or not. Behind our individual preferences lies a world of shared expectations about comfort and fun. Hence, we claim to understand what it means that mobility needs to be fun.

Consumer law is a derivative of private law, created to protect persons in private law relations that are in a dependent position. The idea is to regulate shortcomings of individual autonomy in cases of independence of one, usually weaker, party. Consumer law relies on rules on the general and specific standards of care, the duties to warn and inform the general principles of proper administration, the requirements of fairness and reasonableness, the doctrine of general terms and conditions, and the idea of fixed compensations and quasi-automatic liability. More and more scholars call for a strategy combining data protection enforcement with consumer law protection[35]. Illegitimate processing actions would then be treated as void under the Unfair Contract Terms Directive[36] and consumer organizations would be seen as new promising actors due to their competence to institute class action.

In passing, we note that there is at present no comprehensive consumer protection law framework in Europe. The existing consumer protection provisions can be found in a multitude of directives; eight directives constitute the core regulation in this field. These directives concern contracts negotiated away from business premises, package travel, unfair terms, timeshares, distance contracts, prices, injunctions, and sale of consumer goods[37]. The consumer law *acquis* is currently under review[38], and there are plans to come up with a more general text, a Consumer Rights Directive, covering generally all contracts, whether or not concluded offline or online, at businesses as well as outside business premises. The Directive aims to put in place clear EU-wide rules covering disclosure of full precontractual information; delivery and passing on risk to the consumer; a single EU-wide cooling off period of 14 calendar days; distance and pressure sales, repairs, replacement, and guarantees; right of withdrawal; a ban on preticked opt-in boxes and hidden charges; and a new black list of unfair contract terms that are prohibited across the EU in all cases, as well as an EU-wide grey list of contract terms deemed to be unfair if the trader does not prove the contrary. The rules also cover protection for mobile- and tele-commerce, new EU-wide protection for online auctions, and specific rules concerning information for consumer rights at point of sale. To complete the picture, a standard set of remedies will be available to a consumer who has bought a faulty product.

Cuijpers is one of the leading data protection experts systematically exploring the added value of consumer law protection for data protection issues. She pinpoints several important features of current consumer law regulation[39]. She notes, for instance, that almost all EU consumer protection directives contain some form of information obligations or consumer rights to access. Examples can be taken from the directive on door-to-door selling[40] and from the directive on electronic commerce[41].

The straightforwardness of consumer law is appealing. Contrary to data protection principles and rights, often thought not to be intelligible, consumer law principles are understood and recognized in practice. Similar principles and legal concepts exist in the U.S.[42]. Data protection can pick up ideas that are common ground in consumer law; for instance, the idea of collective action as opposed to data protection rights as individual rights, the idea of generous compensation as opposed to finding damage when breaching data protection, and the idea of security and privacy-enhancing technology as a service. In more general terms, data protection can learn a concern for pragmatism.

A good solution, so we believe, is to introduce more (explicitly) data protection–oriented consumer rights in consumer law. We see no reason why this more general field of law has to remain ignorant of the data protection requirements[43]. The actual review of the EU *acquis* on consumer protection misses the opportunity to embrace data protection concerns. Also, in the proposals for a new directive on consumer rights, no separate attention is paid to privacy and data protection concerns. These matters are referred to the existing EU data protection directives. On the other hand, we see that privacy is one of the keywords on the EU eYouGuide site[44]. When scrolling through this public-oriented guide to consumer rights, it is striking to note how many of the other keywords also strengthen data protection principles.

Combining strengths of the two regimes will certainly benefit the goals of data protection. Take, for instance, the regulation of redress in the EU directives and the way evidence regimes are conceived. The sales of goods directive offers a set of interesting solutions[45]. First, it provides for a “duty of conformity”. Then, in case of nonconformity, the directive assigns to the sellers the burden of proof, while it grants to consumers, without charges, a right to repair and replacement. However, next to the redress mechanism offered to the consumer, the directive also provides for a redress procedure open to the final seller. Indeed, if the nonconformity results from the responsibility of another person in the contractual chain, the final seller is entitled to pursue remedies against him or her. Imagine a similar mechanism in the case of errors occurring with PNR data. A passenger is refused access to a plane. Where is the error? In the data sent to the U.S. by the air company? In the no-fly list kept by the Department of Homeland Security? In the profiling exercise done by the FBI? Instead of leaving it to the single traveler to sort out the specific responsibilities, a one-solution remedy and a generous compensation regime are the least that can be expected.

In practice, this would imply the right for the data subject to turn to every element of the chain rather than to look for the precise defecting element. When a violation of data protection principles is discovered, the choice of a data subject is not limited to any particular actor in a given information chain. The burden of finding the right actor to bring an action against is thus removed from the data subject. An action could be brought against any actor involved to any degree with the piece of data in question if it is not clear where exactly the data regime was violated and which actor was “at fault” or against the actor who was “caught” using personal data in question without proper authorization. The burden to ensure that data transfers occurred without violations would lie on each and every actor “in the cloud” or “in the chain”, and so would the accountability in general and liability for damages. This could be of special relevance in the context of the transborder data transfers to the third countries. While the contemporary discourse is occupied with discovering better ways to ensure a desired level of data protection beyond the EU borders, *inter alia*, by means of binding corporate rules, etc., the “propertization” solution may offer a ready answer. Regardless of the arrangements between the EU and non-EU actors, a data subject in the case of a violation of his/her data protection rights may address his/her claim to the non-EU actor or the EU-based actor, letting him sort out the burden of responsibility with the non-EU counterpart. After paying the damages, the actor in question would have a chance to look further “down the chain” for the source of the violation.

## CONCLUSION

This is not to say that a consumer-friendly approach should fully substitute an effective administrative and judicial redress procedure. On the contrary, what we believe is that a new system should be designed as an efficient complement to the latter, especially given the burden that "traditional" redress procedures engender for individuals, both in terms of time and costs[46].

We also believe that introducing generous compensation and a one-solution remedy would provide important tools to improve the accountability of states' agencies "guess-work" activities. First, because it would create a sort of public "trace" of error rates, attracting more than statistical attention both at administrative and political levels. Second, because states' agencies would no more be confronted merely to single out mistakenly stopped travelers, but also with more organized air companies, which would have a major interest in identifying and highlighting responsibilities and systems' bias.

Also, other ideas are worthy to be further explored and discussed. Many of them have been already presented in public *fora*, and we want, at least, to evoke them: the extension of mandatory data breach notification to all sectors, the integration of elements drawn from the unfair contract terms directive into new privacy notices, the establishment of central points providing assistance to consumers, the possibility for collective action[47].

A good idea of the direction to go is provided by the EU passenger rights. These rights are established by the EU directive on air passenger rights (Regulation 261/2004)[48]. The purpose of this legal instrument is to ensure a high level of protection for passengers. "Moreover, full account should be taken of the requirements of consumer protection in general" (Preamble, sub (1)). The Regulation notes that denied boarding and cancellation or long delay of flights cause serious trouble and inconvenience to passengers (Preamble, sub (2)), and that the number of passengers denied boarding against their will remains too high, as does that affected by cancellations without prior warning and that affected by long delays (Preamble, sub (3)). The core of the Regulation consists in creating new subjective rights for passengers with an effective compensation scheme; the obligations that were created by the Regulation rest with the operating air carrier who performs or intends to perform a flight, "whether with owned aircraft, under dry or wet lease, or on any other basis." Of course, nothing prevents the operating air carrier to seek compensation from any person, including third parties, in accordance with the law applicable.

The effectiveness of the scheme has been illustrated by the air travel problems due to the volcanic ash cloud produced by an Icelandic volcano in April 2010. An EU press release reminded the many passengers that "all the EU passenger rights do apply"[49]:

- The right to receive information from airlines (e.g., on your rights, on the situation as it evolves, cancellations, and length of delays);
- The right to care (refreshments, meals, accommodation as appropriate);
- The right to choose between reimbursement of fares or be rerouted to final destination.

The press release also contained the following disclaimer: "However: In an exceptional circumstance such as this, passengers are <u>not</u> however entitled to additional financial compensation that would be the case where delays or cancellations are the fault of the airline."

The pragmatism of this reservation is evident. Consumer law protection is about correcting power relations. Massive compensation claims directed against airlines can distort the market and impede *too much* on the interests of the stronger parties concerned. We would like to see included in the Regulation a section on the no-fly list where rights and duties, after having been duly balanced, are clearly stated in plain language.

To conclude, we would like to note that civil liberty issues should not be addressed through the lens of consumer law protection only. On the contrary, we are consumers **and** citizens, and the latter position requires a different debate and a different trading act[50]. From a consumers' point of view, it might be feasible to smooth off totalitarian practices, while a genuine citizens' debate on the political merits of these practices would have resulted in the total abandonment of them. Our plea should also not be understood as one in favor of relinquishing specific data protection rules and relying solely on the general contract and consumer protection instruments. Instead, we believe that a mutual reinforcement of both instruments, and a more consumer-friendly approach to border check technology, can foster a citizens' debate freed from the paradoxes of excessive and low visibility.

## ACKNOWLEDGMENT

The authors would like to thank Rosamunde van Brakel for her valuable comments and inputs on a draft version of this article, and Paul Quinn for his precious linguistic advice.

## NOTES

1. Iggy Pop (1977) The passenger. In *Lust for Life.* Thousand Mile, Inc.
2. To some extent, both problems subsume a sort of "essentialist" approach to technology, which "holds that there is one and only one 'essence' of technology and it is responsible for the chief problems of modern civilization." Andrew Feenberg (1999) *Questioning Technology.* Routledge, London/New York. 3.
3. We borrow the concept of "sorting" from David Lyon's important insights on "social sorting". David Lyon (2003) Surveillance as social sorting: computer codes and mobile bodies. In *Surveillance as Social Sorting. Privacy, Risk and Digital Discrimination*. Routledge, London. It should also be noted that the "technological mode" does not fully substitute the more traditional operations of the government of mobilities, such as those that "sort countries" by establishing lists of countries whose citizens should submit to specific, enhanced, controls and restrictions. As discussed elsewhere, the technological mode articulates itself upon the other, partially reshaping its scope (for example, when the access to a privileged list of countries is linked to the acceptation of data sharing agreements).
4. We have discussed the impact of the "routine" use of smart and soft surveillance technologies, such as large biometric databases, on the presumption of innocence and the risk of stigmatization in Rocco Bellanova and Paul De Hert (2009) Le Cas S. Et Marper Et Les Données Personnelles: L'horloge De La Stigmatisation Stoppée Par Un Arrêt Européen. *Cultures & Conflits* 76.
5. "The US currently deploy approximately 200 Security Scanners in 41 airports (...). More unites will be deployed in 2010 and 2011. By 2014 the US plans to have procured and deployed 1800 Security Scanners in order to be able to introduce them as a primary screening method rather than as a secondary screening method or only for alarm resolution." European Commission (2010) Communication from the Commission to the European Parliament and the Council on the Use of Security Scanners at EU Airports. European Commission, Brussels. 9.
6. Ibid., 3. It should be noted that the U.K. government had already started the trial of body scanners before 2010, but this program was further extended and gained increased public attention especially since the beginning of 2010. cf. Home Affairs Committee House of Commons (2010) Counter-Terrorism Measures in British Airports. House of Commons, London.
7. European Commission (2010) Communication from the Commission to the European Parliament and the Council on the Use of Security Scanners at EU Airports. European Commission, Brussels.
8. European Parliament (2008) European Parliament Resolution of 23 October 2008 on the Impact of Aviation Security Measures and Body Scanners on Human Rights, Privacy, Personal Dignity and Data Protection. European Parliament, Strasbourg.
9. Ibid.
10. European Commission (2009) Public Consultations. The Impact of the Use of Body Scanners in the Field of Aviation Security on Human Rights, Privacy, Personal Dignity, Health and Data Protection. http://ec.europa.eu/transport/air/consultations/2009_02_19_body_scanners_en.htm .
11. European Commission (2010) Communication from the Commission to the European Parliament and the Council on the Use of Security Scanners at EU Airports. European Commission, Brussels.
12. A thorough discussion of the impact of the body scanners on human rights surely deserves much more attention and space than the few lines we can devote in the context of this article. First, the number of rights called into question is particularly wide: privacy; data protection; human dignity; the right to equal treatment and nondiscrimination; freedom of thought, conscience, and religion; and physical integrity. cf. Article 29 Data Protection Working Party and European Data Protection Supervisor - EDPS (2009) Consultation. The Impact of the Use of Body Scanners in the Field of Aviation Security on Human Rights, Privacy, Personal Dignity, Health and Data Protection. Data Protection Working Party, Brussels; European Union Agency for Fundamental Rights - FRA (2010) The Use of Body Scanners: 10 Questions and Answers. FRA, Vienna. Second, the controls and searches at the airport are becoming an important element of comparison when discussing other forms of police powers. A recent case of the European Court of Human Rights (ECHR) is particularly telling and challenging. The issue at stake was the compatibility of the "stop and search" powers granted by the U.K. Terrorism Act 2000 with the ECHR and in particular with Article 5, concerning the deprivation of liberty; Article 8, on the respect of private life; Article 10, on the freedom of expression; and Article 11, on the freedom of peaceful assembly and association with others. In its judgment, the ECHR refused the reasoning of the U.K. Government, insisting on the “analogy drawn with the search to which passengers uncomplainingly submit at airports or at the entrance of public buildings,” because “[a]n air traveler may be seen as consenting to such a search by choosing to travel.” *Case of Gillan and Quinton V. The United Kingdom*, §64 (2010). The response of the ECHR seems particularly clear, as well as its view concerning *in situ* airport searches: the air traveler “knows that he and his bags are liable to be searched before boarding the aeroplane and has a freedom of choice.” *Gillan & Quinton*, §64. Nevertheless, its application to the use of body scanners, as well as the validity of consent in such situations, would require a more in-depth analysis on the case law and the specific implementation of this technology. Finally, the comparison between the assessment of “stop and search” police powers and the use of body scanners could also become very useful when discussing the possible questioning of their use in terms of protection against inhuman or degrading treatment or punishment (Article 3 of the European Convention) and the right of not been deprived of liberty (Article 5). However, it should be said that it seems particularly unlikely that a lawyer would use Article 3, which implies physical heavy limitations of the body integrity.

13. It is particularly telling that the 2010 European Commission remains very generic in this respect: “Although questions were raised whether Security Scanners would have been able to prevent the Detroit incident of 25 December 2009, it is clear that given the technology at hand today, the Security Scanners would have maximised the probability to detect the threats and will provide us with a considerably enhanced prevention capability.” European Commission (2010) Public Consultations. The Impact of the Use of Body Scanners in the Field of Aviation Security on Human Rights, Privacy, Personal Dignity, Health and Data Protection. A 2010 report of the U.K. House of Commons states that the scanners deployed in the U.K. have an efficiency rate of 50–60%. cf. House of Commons (2010) Counter-Terrorism Measures in British Airports.
14. One of the main findings of the EPIC FOIA requests was that, despite official declarations on the automatic deletion of images, some of these images are, indeed, stored by federal authorities. cf. Electronic Privacy Information Center - EPIC (2010) Whole Body Imaging Technology and Body Scanners ("Backscatter" X-Ray and Millimeter Wave Screening). http://epic.org/privacy/airtravel/backscatter/.
15. European Commission (2010) Public Consultations. The Impact of the Use of Body Scanners in the Field of Aviation Security on Human Rights, Privacy, Personal Dignity, Health and Data Protection."
16. Also, it is a pity that many contributions to the Commission public consultation are still not available to the public.
17. Formally, no official decision has been taken at the moment of drafting this article, but according to the press, the tests carried on in three Italian airports have been already stopped, and the final decision will be taken only after the formal end of the last test in the fourth airport. cf. Fiorenza Sarzanini (2010) Via I Body Scanner Costati Due Milioni. *Corriere della Sera*, 23.09.2010.
18. Ibid.
19. “Privium is Schiphol’s service program for frequent flyers who wish to travel without unnecessary delay. Privium membership makes travel a pleasant experience for you. (…) Privium offers fast and safe border passage based on modern iris identification technology.” Schiphol Airport. Why Privium? http://www.schiphol.nl/Travellers/AtSchiphol/PriviumIrisscan/WhyPrivium.htm.
20. Or, in the words of Schiphol Airport: “Every eye is unique, which makes an iris scan as reliable as passport control. Sophisticated identification equipment is used to ‘read’ your eye. The procedure is completely harmless and poses no risk whatsoever to your eyes or health. Plus, compared to other types of biometric identification, it is the fastest and most reliable. All you need is a Privium Card.” In any case, it is important to note that the iris scan reading is not always possible, nor the initial collection of data.
21. Such a practice of creation of a “trusted traveler” status is a particularly evident case of the already discussed diffusion of practices of “social sorting”, whose goal is to sift individuals and cluster them in classes that are “marketed” in tailored ways. cf. Lyon, "Surveillance as Social Sorting: Computer Codes and Mobile Bodies."
22. In 2008, the Commission presented a Communication on the next steps of EU border management, including, *inter alia*, the idea of a EU-wide “travellers registered program” open to both third-country nationals and EU citizens. Also in this case, the idea implies the use of biometrics to “smooth” the passage at automated border gates (and the possibility to use this automated port of entry was presented as the main incentive for EU citizens). cf. European Commission (2008) Communication from the Commission to the European Parliament, the Council, the European Economic and Social Committee and the Committee of the Regions. Preparing the Next Steps in Border Management in the European Union. European Commission, Brussels. 6–7. It should be noted that all the measures presented in 2008, including the trusted travelers program, remain in the Stockholm Programme.
23. European Data Protection Supervisor - EDPS. Glossary. Biometrics. http://www.edps.europa.eu:80/EDPSWEB/edps/EDPS/Dataprotection/Glossary/pid/72.
24. Cf. Pierre Piazza (2005) Alphonse Bertillon Face À La Dactyloscopie. Nouvelle Technologie Policière D’identification Et Trajectoire Bureaucratique. *Cah. Sécurité Intérieure*. 56; S.A. Cole (2002) *Suspect Identities: A History of Fingerprintings and Criminal Identification.* Harvard University Press, Cambridge.
25. Institute for Prospective Technological Studies (2005) Biometrics at the Frontiers: Assessing the Impact of Society. European Commission, Seville. 31–39.
26. As said before, one of the main goals of the introduction of the biometric passport was the strengthening of the passport security (and the facilitation of the processes of verification). The EC Regulation does not provide for limits on the validity of the new passport or on a common price. Both decisions are left to Member States. It should be noted that, in general, the prices augmented and that different versions of the same national passport were created every few years to both implement EU requirements as well as those of the U.S.

27. Error rates include both false positive and false negative. Next to them, the case of inability to collect (or verify after the first collection) biometrics for physical reasons should also be added.
28. Paul De Hert (2005) Biometrics: Legal Issues and Implications. Background Paper for the Institute of Prospective Technological Studies, DG JRC – Sevilla, European Commission.
29. In the U.S., this notion is clearly stated in the text of the 4th Amendment of the U.S. Bill of Rights: “The right of the people to be secure in their persons, houses, papers, and effects, against unreasonable searches and seizures, shall not be violated; and no Warrants shall issue but upon probable cause, supported by Oath or affirmation, and particularly describing the place to be searched, and the persons or things to be seized.”
30. Article 5(5) of the European Human Rights Convention states: “Everyone who has been the victim of arrest or detention in contravention of the provisions of this article shall have an enforceable right to compensation.”
31. Cf. the relatively vague description offered by the 2010 Commission Communication on a global approach to PNR transfer, and the more complete description of the PNR processing schemes provided in the U.S. System of Record Notification of the Automated Targeted System. European Commission (2010) Communication from the Commission. On the Global Approach to Transfers of Passenger Name Record (PNR) Data to Third Countries. European Commission, Brussels. 3–4; DHS Privacy Office (2007) Notice of Privacy Act System of Records. U.S. Customs and Border Protection, Automated Targeting System. Department of Homeland Security, Washington, D.C.
32. On the U.S. application of the EU-US PNR agreement, cf., *in extenso*, European Commission (2010) Report on the Joint Review of the Implementation of the Agreement between the European Union and the United States of America on the Processing and Reansfer of Passenfer Name Record (PNR) Data by Air Carriers to the United States Department of Homeland Security (DHS). 8–9 February 2010. European Commission, Brussels.
33. The U.S. Department of Homeland Security has introduced, back in 2009, an Electronic System of Travel Authorization (ESTA), which obliges the citizens of the Visa Waiver Program countries to complete electronically, and in advance of at least 72 h, a detailed questionnaire. The form is the same as the paper questionnaire to be completed at the ports of entry, but its electronic format permits the DHS to run the information against other databases and start border checks in advance. Apparently, the ESTA system could provide a consumer-friendly solution, however, two important elements strongly limit this potential. First, the “green light” received by the ESTA system does not grant any right to effectively enter the U.S., and it can also be reversed by a different outcome of other systems run on the same passenger data. Second, after a first period of trial in which ESTA applications were free of charge, a fee of $14 has been imposed to passengers. cf. Department of Homeland Security (2008) Privacy Impact Assessment for the Electronic System for Travel Authorization (ESTA). Department of Homeland Security, Washington, D.C.; Valentina Pop (2010) US Travel Fee Draws New Dividing Line with EU. *euboserver.com*, 24.09.2010.
34. On compensation for those who are flagged erroneously, see Gloria González-Fuster and Paul De Hert (2007) PNR and compensation. In *Are You Who You Say You Are? The EU and Biometric Borders*. Lodge, J., Ed. Wolf Legal Publisher, Nijmegen.
35. Colette Cuijpers (2007) A Private Law Approach to Privacy; Mandatory Law Obliged? *SCRIPT-ed* 4(4); Jan Berkvens (2009) Role of trade associations: data protection as a negotiable issue. In *Reinventing Data Protection?* Gutwirth, S. et al., Eds. Springer, Dordrecht. 128.
36. Directive 1993/13/EC
37. See below for a discussion of some of these Directives. See also Council Directive 93/13/EEC of 5 April 1993 on unfair terms in consumer contracts, *Official Journal*, L 95, 21 April 1993, p. 29–34; Directive 1999/44/EC of the European Parliament and of the Council of 25 May 1999 on certain aspects of the sale of consumer goods and associated guarantees, *Official Journal*, L 171, 7 July 1999, p. 12–16; Directive 97/7/EC of the European Parliament and of the Council of 20 May 1997 on the protection of consumers in respect of distance contracts, *Official Journal*, L 144, 4 June 1997, p. 19–27; Council Directive 85/577/EEC of 20 December 1985 to protect the consumer in respect of contracts negotiated away from business premises, *Official Journal*, L 372, 31 December 1985, p. 31–33. See in general: Stephen Weatherill (2005) EU Consumer Law and Policy. Elgar European Law, Northampton. 1–33; Colette Cuijpers (2009) The Influence of ICT on Consumer Protection; Empowerment or Impairment of the Consumer? In Tilburg University Legal Studies Working Paper.
38. Green Paper on the Review of the Consumer Acquis, COM (2006) 744 Final
39. See also Colette Cuijpers and Bert-Jaap Koops (2008) How fragmentation in European law undermines consumer protection: the case of location-based services. *Eur. Law Rev.* 33.
40. Article 5 of the Directive on door-to-door selling requires traders to give consumers written notice of their right of cancellation of a concluded contract, together with certain information such as the name and address of the person against whom that right may be exercised. cf. Council Directive 85/577/EEC of 20 December 1985 to protect the consumer in respect of contracts negotiated away from business premises, *Official Journal*, L 372, 31 December 1985, p. 31–33; Colette Cuijpers (2009) The Influence of ICT on Consumer Protection; Empowerment or Impairment of the Consumer? In Tilburg University Legal Studies Working Paper.
41. Directive 2000/31/EC of the European Parliament and of the Council of 8 June 2000 on certain legal aspects of information society services, in particular electronic commerce, in the Internal Market (Directive on electronic commerce), *Official Journal*, L 178, 17 July 2000, p. 1–16. In particular, the Directive on electronic commerce includes several provisions on information obligations: "[a]rticle 5 concerns general information to be provided by service providers (…). Article 10 supplements the information obligation for service providers in relation to the conclusion of electronic contracts. In this respect several categories of information are identified that the service provider must offer consumers in a clear, comprehensible and unambiguous manner, prior to an order being placed by the recipient of the service. Another kind of information obligation concerns the acknowledgement of receipt of the recipient's order as regulated in article 11. In respect of directories of subscribers, Article 12 provides for subscribers to be informed, free of charge and before they are included in the directory, about the purpose(s) of a printed or electronic directory and of any further usage possibilities based on search functions embedded in electronic versions of the directory. Subscribers must be given the opportunity to have some control over their personal data and they must be able to verify, correct or withdraw such data"; Ibid., 11.

42. For a short discussion of U.S. consumer law: Ibid., 15 and following.
43. On the idea that data protection aspects are ignored when drafting consumer legislation, cf. Colette Cuijpers and Bert-Jaap Koops (2008) How fragmentation in European law undermines consumer protection: the case of location-based services. *Eur. Law Rev.* 33.
44. http://ec.europa.eu/information_society/eyouguide/index_en.htm
45. Directive 1999/44/EC of the European Parliament and of the Council of 25 May 1999 on certain aspects of the sale of consumer goods and associated guarantees, *Official Journal*, L 171, 7 July 1999, p. 12–16.
46. During a seminar held in 2010 in Madrid, on the issue of Security, Privacy and Data Protection, an American expert underlined the poor possibilities to obtain redress (and even access information) in cases linked to the processing of PNR or inscription to no-fly lists. cf. David L. Sobel (2010) EU-U.S. Data Sharing: The Limitations of Redress. Paper Presented at the Seminar on Security, Privacy and Data Protection, Madrid, 09.06.2010. Electronic Frontier Foundation, Washington, D.C.
47. These and other ideas and suggestions were presented by Willemien Bax at the Data Protection Conference organized by DG Justice, Freedom and Security, European Commission, on 19–20 May 2009; Willemien Bax (2009) Consumer Protection and Personal Data Protection. BEUC, Brussels. Speech. cf. also, http://webcast.ec.europa.eu/eutv/portal/archive.html?viewConference=7334&catId=7256
48. Regulation (EC) No 261/2004 of the European Parliament and of the Council of 11 February 2004 establishing common rules on compensation and assistance to passengers in the event of denied boarding and of cancellation or long delay of flights, and repealing Regulation (EEC) No 295/91, *Official Journal* L 046, 17 February 2004, p. 1 -8.
49. http://europa.eu/rapid/pressReleasesAction.do?reference=MEMO/10/283&type=HTML
50. See on these different positions: Cass Sunstein (2007) *Republic.Com 2.0.* Princeton University Press, Princeton.
